# Differential infection behavior of African swine fever virus (ASFV) genotype I and II in the upper respiratory tract

**DOI:** 10.1186/s13567-023-01249-8

**Published:** 2023-12-15

**Authors:** Dayoung Oh, Shaojie Han, Marylène Tignon, Nadège Balmelle, Ann Brigitte Cay, Friso Griffioen, Brecht Droesbeke, Hans J. Nauwynck

**Affiliations:** 1 Laboratory of Virology, Department of Translational Physiology, Infectiology and Public Health, Faculty of Veterinary Medicine, Ghent University, Merelbeke, Belgium; 2 Department Infectious Diseases in animals, Service of Viral Reemerging Enzootic and Bee Diseases, Sciensano, Groeselenberg 99, Brussels, Belgium

**Keywords:** African swine fever virus, genotype I, genotype II, nasal mucosa, porcine respiratory epithelial cells, intercellular junctions

## Abstract

African swine fever virus (ASFV) is a substantial threat to pig populations worldwide, contributing to economic disruption and food security challenges. Its spread is attributed to the oronasal transmission route, particularly in animals with acute ASF. Our study addresses the understudied role of nasal mucosa in ASFV infection, using a nasal explant model. The explants remained viable and revealed a discernible ASFV infection in nasal septum and turbinates post-inoculation. Interestingly, more infected cells were found in the turbinates despite its thinner structure. Further analyses showed (i) a higher replication of genotype II strain BEL18 than genotype I strain E70 in the epithelial cell layer, (ii) a preference of ASFV infection for the lamina propria and a tropism of ASFV for various susceptible cell types in different areas in the nasal mucosa, including epithelial cells, macrophages, and endothelial cells. Using porcine respiratory epithelial cells (PoRECs), isolated from nasal tissue, we found a difference in infection mechanism between the two genotypes, with genotype I favoring the basolateral surface and genotype II preferring the apical surface. Moreover, disruption of intercellular junctions enhanced infection for genotype I. This study demonstrated that ASFV may use the respiratory mucosa for entry using different cell types for replication with a genotype difference in their infection of respiratory epithelial cells.

**Supplementary Information:**

The online version contains supplementary material available at 10.1186/s13567-023-01249-8.

## Introduction

African swine fever (ASF) is a highly infectious disease that impacts both domestic pigs and wild boars, with a potential lethality rate up to 100% [1]. The etiological agent, ASF virus (ASFV) is a large double-stranded DNA virus and the only member of the family *Asfarviridae* [2]. ASFV is classified into 24 genotypes based on sequencing of the variable region of the B646L gene, which encodes the major capsid protein, p72 [3]. Since the disease was described in 1920s, all identified genotypes persist in Africa. The first detection of ASFV outside Africa happened in 1957, when genotype I was reported in Lisbon, Portugal. This marked the beginning of its spread to neighboring countries in Europe and Latin America [4, 5]. A second escape of ASFV from Africa took place in 2007 when genotype II was reported in Georgia [6]. This genotype exhibits a high degree of virulence and is currently driving a widespread pandemic in Europe, Asia, Africa, and the Caribbean islands (source: WOAH report, March 17–30, 2023) [7]. Since there is no vaccine registered for ASFV, except in Vietnam [8], knowledge and countermeasures against the disease have become top veterinary priorities, considering the disease’s economic impact, its threat to pork product supplies, and issues with animal welfare.

ASFV can be transmitted through various routes, including oronasal ingestion, aerosol exposure, iatrogenic, via semen, and through soft ticks (biological reservoir) [9, 10]. Affected pigs release the virus into their surroundings via secretions and excretions, including oral and nasal fluids, blood, feces, and urine. These contain a high concentration of virus during the disease’s acute phase. Direct nose-to-nose contact or inhalation of viruses present in aerosols can increase the risk of viral transmission. It has been shown that intranasally inoculated pigs develop acute disease and virus can be detected in nasal swabs of the air-contact pigs [11]. This suggests that infectious ASFV particles are present in the nasal mucosa. However, ASFV pathogenesis in the nasal mucosa remains unknown.

The nasal mucosa is the first tissue that allows pathogens entering the respiratory system. The outermost layer is the pseudostratified ciliated columnar epithelium, whose integrity and polarity are meticulously maintained by intercellular junctions (ICJ) [12]. Beneath lies the lamina propria, a loose connective tissue layer populated with glands and immune cells [13, 14]. The submucosa, similar but denser than the lamina propria, resides at a deeper level [15]. Epithelial cells in the nasal airway are often first targets for viruses, followed by immune cells in the epithelium and lamina propria [16]. These infected immune cells allow viruses penetrating the epithelial barrier, navigating through the lamina propria, and eventually entering the bloodstream [17, 18].

In this study, we employed a respiratory mucosal explant model that mimics in vivo conditions to explore the infection patterns of ASFV in the nasal mucosa, using genotype I strain E70 and genotype II strain Belgium 2018/1 (BEL18). Additionally, we isolated and cultivated primary porcine respiratory epithelial cells (PoRECs) on transwells to examine the polarity of ASFV binding and subsequent viral replication.

## Materials and methods

### Nasal explant and PoRECs culture

Six 3- to 4-week-old healthy crossbred pigs (Landrace x Pietrain) were used in this study; three were used for the production of nasal explants; three were used for the production of porcine respiratory epithelial cell (PoRECs) cultures. After intravenous euthanasia with pentobarbital (Kela, Hoogstraten, Belgium) at 12.5 mg/kg body weight, nasal septum and turbinates were collected. Isolation and culture of nasal explants were adapted from the previously described method [19]. Briefly, nasal septum and turbinate were collected by carefully stripping off from the bone. Mucus was washed on ice-cold transport medium containing HBSS with calcium and magnesium (Gibco, Paisley, UK), 0.1 mg/mL gentamicin (Gibco, Paisley, UK), 0.1 mg/mL streptomycin (Gibco, Grand Island, NY, USA), 100 U/mL penicillin (Gibco, Grand Island, NY, USA), and 5 μg/mL amphotericin B (Gibco, Grand Island, NY, USA). Tissues were then cut in small square pieces (25 mm^2^) and immediately placed epithelial side facing up on a find-meshed grid in a 6-well plate containing serum-free medium containing DMEM/RPMI 1640 (Gibco, Paisley, UK) (1:1 ratio), 0.1 mg/mL gentamicin, 0.1 mg/mL streptomycin, 100 U/mL penicillin. The explants were cultivated at an air–liquid interface for 24 h (37 °C, 5% CO_2_).

Isolation and culture of PoRECs were adapted from the previously described method for the isolation of equine respiratory epithelial cells (ERECs) [20]. Nasal septum and turbinate tissues were pooled and incubated with an enzyme mix of 1.12 mg/mL pronase (Roche Diagnostics, Mannheim, Germany) and 80 μg/mL DNase I (Roche Diagnostics, Mannheim, Germany) in calcium- and magnesium-free PBS supplemented with 25 mM glucose (VWR international, Leuven, Belgium), 1% sodium pyruvate (Gibco, Paisley, UK), 0.1 mg/mL streptomycin, 100 U/mL penicillin, and 5 μg/mL amphotericin B for 24 h at 4 °C. Detached cells were then incubated in DMEM/F12 (1:1 ratio) containing 1% non-essential amino acids (NEAA) (Gibco, Paisley, UK), 0.12% Insulin-Transferrin-Selenium-Ethanolamine (ITS-X) (Gibco, Grand Island, NY, USA), 0.1 mg/mL streptomycin, 100 U/mL penicillin, and 5 μg/mL amphotericin B in a cell-culture petri dish for 4 h to reduce fibroblast contamination by adherence. Isolated PoRECs were analyzed by trypan blue staining and seeded at a density of 0.8 million cells per transwell-insert in a 12-well plate and cultivated overnight in Afi1 medium which contains DMEM/F12 (1:1 ratio), 5% fetal bovine serum (FBS) (Sigma-Aldrich, St. Louis, MO, USA), 1% NEAA, 0.1 mg/mL streptomycin, 100 U/mL penicillin, and 5 μg/mL amphotericin B. Transwell inserts with 1.0 μm pore size (Falcon) were used and pre-coated with type IV collagen (Sigma-Aldrich, St. Louis, MO, USA). Cells were then washed with DMEM/F12 medium and replaced by Afi2 medium which contains 2% Ultroser G (Sartorius, Cergy, France) instead of FBS in the Afi1 medium. The medium was added to the bottom well to have an air–liquid interface and the cells were cultivated for 5–7 days.

### Virus inoculation

In this study, two genotypes of ASFV strain were used: a third passage of genotype I strain E70 (Spanish isolate obtained in 1970) in porcine alveolar macrophages (PAMs) and a third passage of genotype II strain Belgium 2018/1 (BEL18, Belgian isolate obtained in 2018 in Etalle, Belgium) in PAMs. After 24 h of pre-incubation, the explants were carefully transferred into a 24-well plate and inoculated with 600 μL of each strain at a titer of 10^6.47^ tissue culture infectious dose 50% endpoint (TCID_50_)/mL suspended in serum-free DMEM/RPMI 1640 medium for 1 h at 37 °C in the presence of 5% CO_2_. After washing with medium, explants were placed back to the gauzes and further incubated in serum-free DMEM/RPMI 1640 medium at air–liquid interface. Explants were collected and embedded in methylcellulose at 0-, 24-, 48-, and 72-h post-inoculation (hpi) and stored at −70 °C.

For PoRECs, Afi2 medium was removed from the bottom well and 200 μL of each strain at 10^6.47^ was inoculated via either the apical or the basolateral surface for 1 h at 37 °C. For the apical side inoculation, cells were pre-treated with 25 mM ethylene glycol-tetra-acetic acid (EGTA) (VWR international, Leuven, Belgium) for 30 min at 37 °C, 5% CO_2_ to see the effect of the disruption of intercellular junctions on ASFV infection. After washing with medium, cells were further incubated in the Afi2 medium and then, fixed at 0, 24, 48, and 72 hpi, with absolute methanol at −20 °C for 30 min. Fixed cells were stored at −20 °C until the following immunofluorescence staining.

### Immunofluorescence microscopy

This study includes three different types of immunofluorescence staining: (1) Explant viability; (2) Identification and characterization of ASFV positive cells in the nasal explants, and (3) Identification and characterization of ASFV positive cells in the PoRECs. The slides were analyzed using an ECLIPSE Ts2R-FL inverted microscope (Nikon, Melville, NY, USA). The detail information of antibodies used for the immunofluorescence staining is illustrated in Table 1.

**Table 1:** **Antibodies used in this study**

Primary antibodies	Clone	Isotype	Working dilution	Supplier
FITC-ASFV major capsid protein p72	1BC11	FITC-conjugated	1:100	Ingenasa
Porcine CD163	2A10	IgG1	1:200	Bio-Rad
Human CD163		Polyclonal	1:200	R&D systems
Porcine CD14	MIL2	IgG2b	1:100	[21]
Porcine SWC3 (CD172a)	DH59B	IgG1	1:50	VMRD
Porcine Sn	41D3	IgG1	1:50	[22]
Human CD1c	L161	IgG1	1:50	Biolegend
Porcine MHCII	MSA3	IgG2a	1:200	Kingfisher Biotech
Human cytokeratin	AE1/AE3	IgG1	1:50	Dako
Porcine vimentin	V9	IgG1	1:50	Bio-Rad
Human vWF		Polyclonal	1:50	Dako

Secondary antibodies (conjugate)	Host/species reactivity	Isotype	Working dilution	Supplier
Alexa Fluor 594	Goat anti-mouse	IgG1	1:400	Invitrogen
Alexa Fluor 594	Goat anti-mouse	IgG2a	1:500	Invitrogen
Alexa Fluor 594	Goat anti-mouse	IgG2b	1:200	Invitrogen
Alexa Fluor 594	Rabbit anti-goat	IgG (H+L)	1:200	Invitrogen
Texas Red	Goat anti-rabbit	IgG (H+L)	1:50	Invitrogen
Alexa Fluor 647	Rabbit anti-goat	IgG (H+L)	1:300	Invitrogen

For explants, cryosections of 9 μm were made and fixed with 4% paraformaldehyde for 15 min at 4 °C and permeabilized in 0.1% Triton-X for 10 min at room temperature (RT). To evaluate the viability of the explants, a terminal deoxynucleotidyl transferase-mediated dUTP nick-end labeling (TUNEL) staining (Roche Diagnostics, Mannheim, Germany) was performed according to the manufacturer’s instructions. The numbers of TUNEL-positive cells and total number of cells in the epithelium, lamina propria, and submucosa were counted separately and transformed into a percentage.

To identify ASFV-positive cells and characterize the infected cells in the nasal explants, sections were incubated for 1 h at 37 °C with FITC-conjugated ASFV major capsid protein p72 (Ingenasa, Madrid, Spain) antibody together with one of the following cell marker antibodies: porcine CD163 (Bio-Rad, Oxford, UK), porcine CD14 [21], porcine SWC3 (CD172a) (VMRD, Pullman, WA, USA), porcine sialoadhesin (Sn) [22], human CD1c (Biolegend, San Diego, CA, USA), porcine MHCII (Kingfisher Biotech, St. Paul, MN, USA), human cytokeratin (Dako, Carpinteria, CA, USA), porcine vimentin (Bio-Rad, Oxford, UK), or human von Willebrand factor (vWF) (Dako, Carpinteria, CA, USA). After washing with PBS, sections were incubated for 1 h at 37 °C with isotype and host species matched Alexa Fluor 594 antibody (Invitrogen) (Table 1). Cell nuclei were counterstained using 5 μg/mL Hoechst 33342 (Invitrogen). The total number of ASFV-positive cells and double ASFV and cell marker positive cells were counted in the epithelium, lamina propria, and submucosa and calculated in percentage.

For the identification of ASFV infection in PoRECs, cells were incubated for 1 h at 37 °C with a FITC-conjugated ASFV p72 in combination with a human cytokeratin antibody followed by secondary antibody incubation for 1 h at 37 °C with a goat anti-mouse IgG1 Alexa Fluor 594 (Invitrogen). For the additional characterization of the infected cells, cells were stained by a triple immunofluorescence with a FITC-conjugated ASFV p72 antibody, a human CD163 (R&D systems, Mineapolis, MN, USA) antibody, and a porcine vimentin antibody. Primary antibodies were diluted in PBS with 10% rabbit serum (Invitrogen) and incubated for 1 h at 37 °C, followed by incubation with a rabbit anti-goat IgG Alexa Fluor 594 (Invitrogen). Then, non-specific binding sites were blocked with 10% goat serum for 30 min at 37 °C. The cells were subsequently incubated with goat anti-mouse IgG1 FITC (Invitrogen) (Table 1). Nuclei were counterstained with Hoechst 33342. The total number of ASFV-positive cells and double- or triple-ASFV and cell marker positive cells were counted and calculated in percentage.

### Statistical analysis

All data were expressed as mean ± standard deviation (SD) from independent experiments from three animals. Statistical analysis was performed using Prism 9 (GraphPad, San Diego, CA, USA). Differences between sample groups were analyzed using two-way analysis of variance (ANOVA) followed by multiple comparison analyses using either Tukey’s or Šidák’s method. *P* value of < 0.05 was considered significant. Additional file 5A was created by Biorender.

## Results

### Evaluation of the nasal explant viability

**Figure 1:**
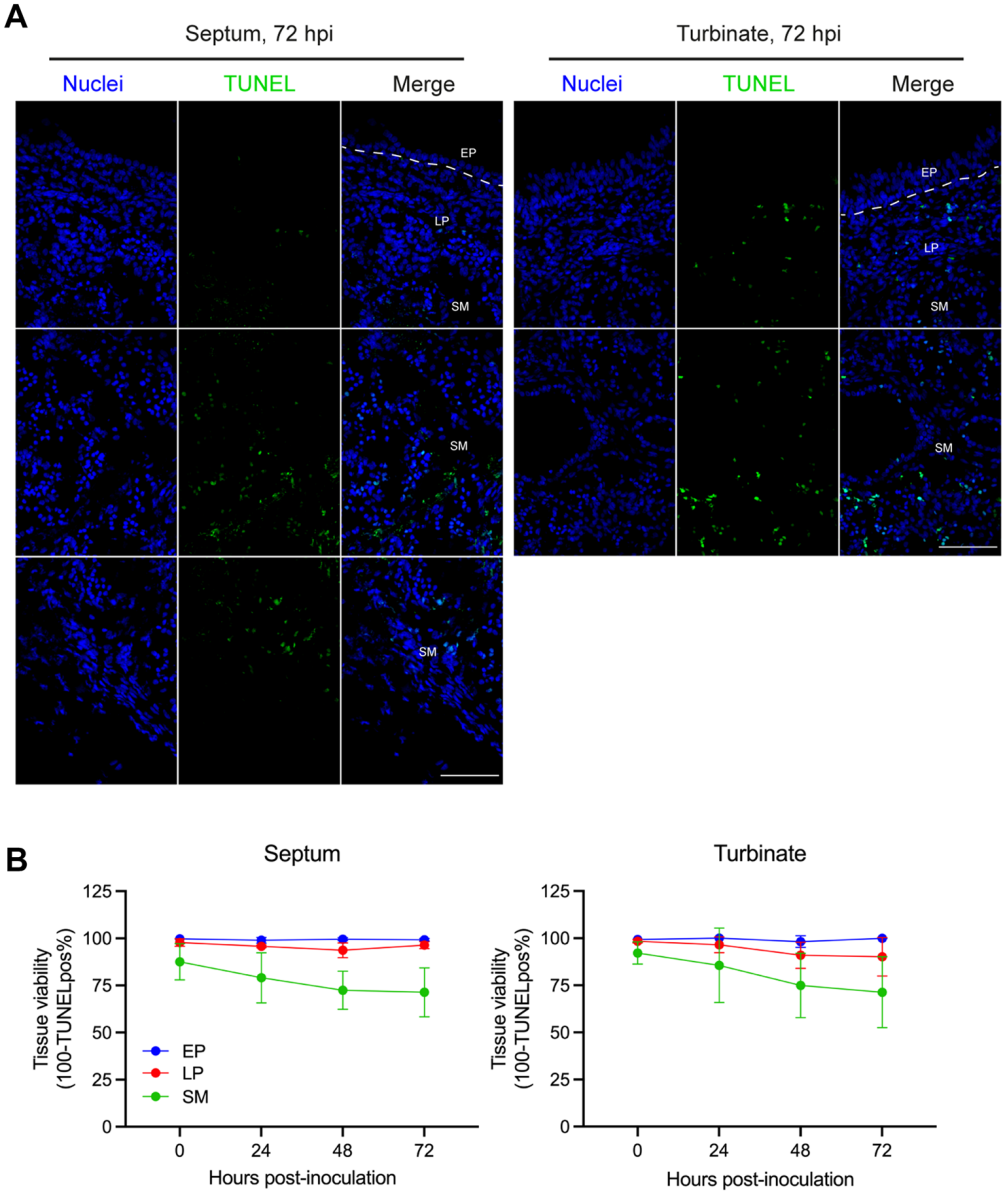
**Evaluation of the nasal explant viability by TUNEL staining at different hours of cultivation.** **A** Representative images of TUNEL staining of the nasal septum and nasal turbinate explants after 72 h incubation. Apoptotic cells (green) and nuclei (blue). White lines indicate the border between the mucosa epithelium and the lamina propria. EP: epithelium, LP: lamina propria, SM: submucosa. Scale bar: 100 μm. **B** Viability of the nasal septum and nasal turbinate explants was assessed in three areas of the nasal mucosa; EP, LP, and SM. Data are presented in mean values ± standard deviation (SD) of three independent experiments.

To assess the viability of nasal mucosa explants upon in vitro cultivation, the percentage of apoptotic cells [terminal deoxynucleotidyl transferase-mediated dUTP nick-end labeling (TUNEL)-positive] was calculated. Mock-inoculated samples from 0, 24, 48, and 72 hpi were analyzed (Figure 1A). Cells in the epithelium (EP) and the lamina propria (LP) showed high cell viability after 72 h incubation (EP-septum: 99.26 ± 0.65%, EP-turbinates: 99.94 ± 0.10%, LP-septum: 96.36 ± 1.83%, and LP-turbinates: 90.19 ± 10.21%) (Figure 1B). Although the number of apoptotic cells increased over incubation time within the submucosa (SM), viable cells were still dominant at 72 hpi (SM-septum: 71.37 ± 12.97% and SM-turbinate: 71.33 ± 18.79%) (Figure 1B).

### Identification of ASFV infection in the nasal explant

**Figure 2:**
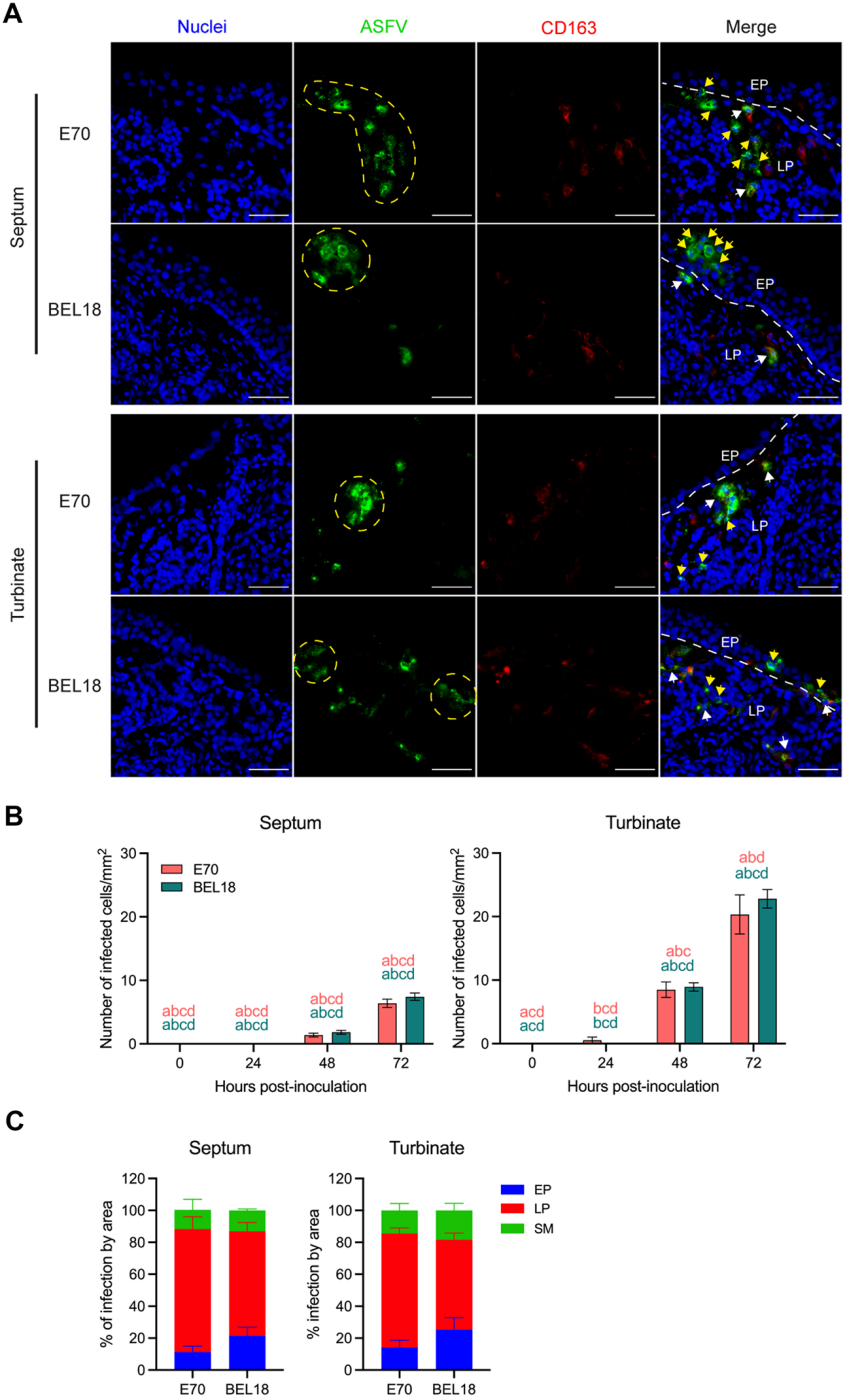
**Identification of ASFV infected cells in the nasal explants.** **A** Double IF staining for ASFV p72 (green) in combination with CD163 (red) was performed on the nasal septum (top) and the turbinate (bottom) explant at 72 hpi. Blue color presents the nuclei staining. The dotted yellow lines present a cluster of infection. The dotted white lines indicate the border between the mucosal epithelium (EP) and the lamina propria (LP). ASFV^+^CD163^+^ cells and ASFV^+^CD163^−^ cells are indicated with white arrows and yellow arrows, respectively. Scale bar: 50 μm. **B** Kinetic study of ASFV infection in the nasal explant. The total number of infected cells in a section from 0, 24, 48, and 72 hpi is presented in mm^2^ area. Statistical significance was determined by two-way ANOVA followed by Tukey’s multiple comparison post hoc test. Different letters represent significant differences (p < 0.05) between time points. All data are presented as mean value of three animals ± standard deviation (SD). **C** Percentage of ASFV infected cells by the areas in the nasal explants. In both nasal tissue types and genotypes, most infected cells were found in the lamina propria. EP: epithelium, LP: lamina propria, SM: submucosa.

The presence of ASFV infected cells was determined in three regions of the nasal mucosa (epithelium, lamina propria, and submucosa), by immunofluorescence (IF) staining against CD163 and ASFV major capsid protein p72 (Figure 2). Infection by genotype I strain E70 and genotype II strain BEL18 was detected in both nasal septum and turbinates. Most of infected cells were found in clusters especially in the epithelium and the lamina propria (Figure 2A). In addition, many infected cells were identified as CD163-negative, especially in the epithelium (Figure 2A). The total number of infected cells increased over incubation time in both nasal tissues inoculated by each strain (Figure 2B). There was no significant difference between E70 and BEL18. More infected cells were found in the turbinate explant at 72 hpi (E70: 20.33 ± 3.07 cells/mm^2^; BEL18: 22.80 ± 1.45 cells/mm^2^) than in the septum explant (E70: 6.38 ± 0.64 cells/mm^2^; BEL18: 7.42 ± 0.59 cells/mm^2^). To study the pattern of infection in different areas of the nasal mucosa, infected cells were quantified and the proportion of infected cells in each area was calculated as percentage (Figure 2C). Infected cells were mainly found in the lamina propria in both septum (E70: 77.08 ± 7.67%; BEL18: 65.48 ± 5.53%) and turbinates (E70: 71.48 ± 3.51%; BEL18: 56.35 ± 4.21%). In both septum and turbinates, infection of E70 was similar between the epithelium (septum: 11.29 ± 3.62%; turbinates: 14.11 ± 4.57%) and the submucosa (septum: 11.98 ± 6.61%; turbinates: 14.42 ± 4.34%). On the other hand, BEL18 showed higher infection in the epithelium (septum: 21.47 ± 5.37%; turbinates: 25.27 ± 7.53%) than in the submucosa (septum: 13.05 ± 0.97%; turbinates: 18.38 ± 4.41%).

### Various types of cells in the nasal mucosa are susceptible to ASFV

Since we detected both CD163-positive and CD163-negative ASFV-positive cells in different regions of the nasal mucosa, further characterization was necessary using the following cell type markers: CD163 and CD14 (marker for monocyte/macrophage), SWC3 (marker for myeloid cell), Sn (marker for macrophage), CD1c (marker for antigen presenting cell), cytokeratin (marker for epithelial cell), vimentin (marker for fibroblast/mesenchymal cell), MHCII (marker for antigen presenting cell), and von Willebrand factor (marker for endothelial cell) (Additional file 1). We first validated the reactivity of the selected markers by performing IF staining on the lungs and nasal tissue sections. The lung section showed positivity to all the selected cell markers, except for the endothelial cell marker, von Willebrand factor (vWF) (Additional files 1 and 2). On the other hand, SWC3 and CD1c were negative in the nasal tissue sections. Thus, these markers were excluded from the further characterization of ASFV infected cells.

**Figure 3:**
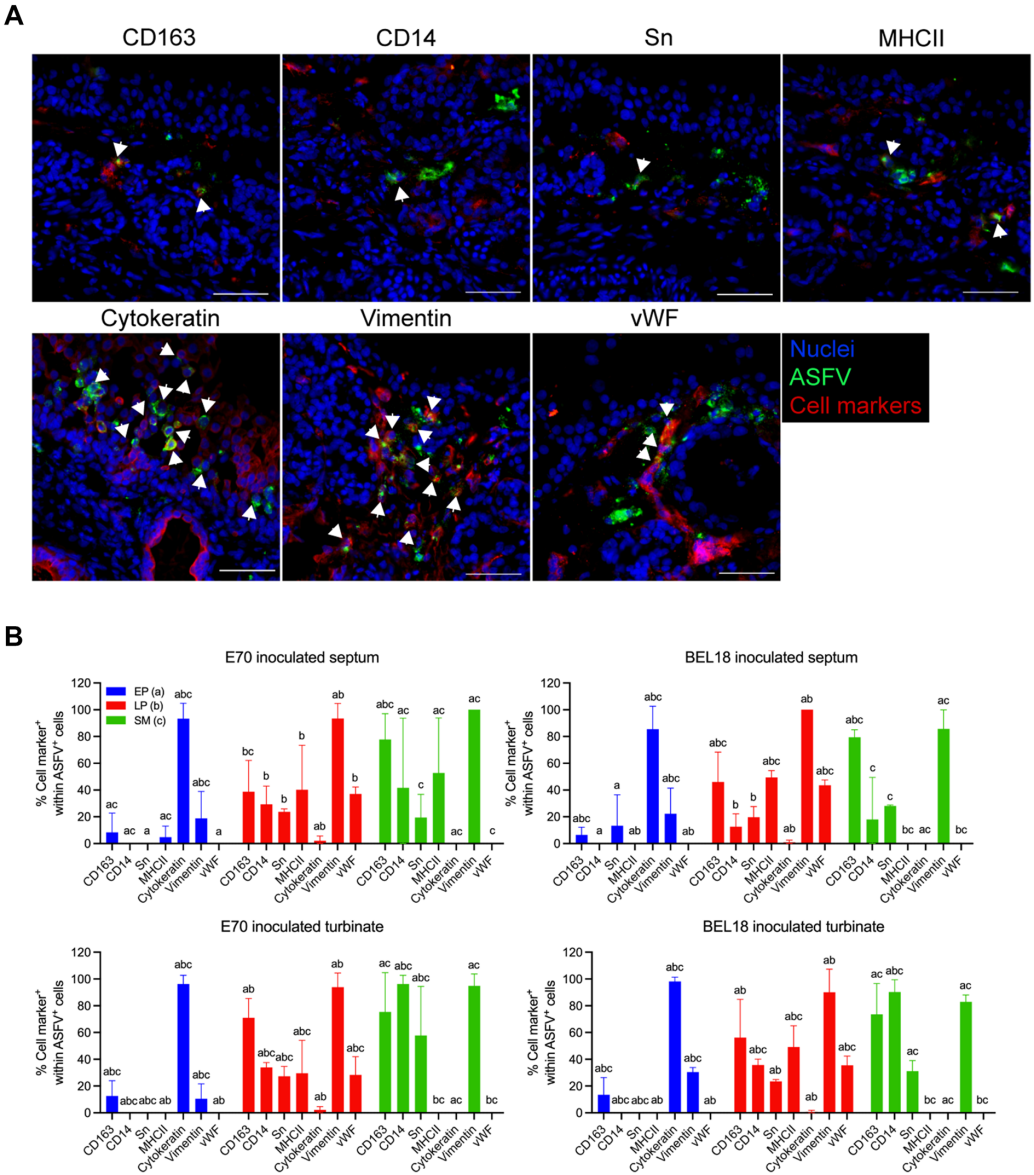
**Characterization of the ASFV infected cells in different areas of the nasal explants.** **A** Double immunofluorescence staining of ASFV (green) together with different cell type markers (red) was performed on the nasal explant. Images were taken from the explants at 72 hpi. Double positive cells are indicated with white arrows. Scale bar: 50 μm. **B** Percentage of cell marker-positive cells within the ASFV-infected cells in each two nasal tissue type inoculated with E70 and BEL18 strains. Statistical significance was determined by two-way ANOVA followed by Tukey’s multiple comparison post hoc test. Different letters represent significant differences (*p* < 0.05) of double-positive cells in different areas of the nasal mucosa: epithelium (EP), lamina propria (LP), and submucosa (SM). All data are presented as mean value of three animals ± SD.

To characterize the phenotype of ASFV infected cells in the nasal explant, we performed double IF stainings against ASFV and the selected cell markers and calculated the proportion of cell marker-positive cells among the infected cells in the epithelium, lamina propria, and submucosa (Figure 3A). Additional file 3 provides the percentage of each cell marker-positive cells within the population of infected cells, in the different areas of the nasal mucosa, two nasal tissue types (septum and turbinates), and two ASFV genotypes (E70 and BEL18). Each group is labeled as septum-E70, septum-BEL18, turbinate-E70, and turbinate-BEL18. In our analysis of both genotypes and types of nasal explants, we observed that a significant fraction of the virus-infected cells within the epithelium were cytokeratin-positive epithelial cells (septum-E70: 93.33 ± 11.55%, septum-BEL18: 85.56 ± 17.11%, turbinate-E70: 96.30 ± 6.42%, turbinate-BEL18: 98.15 ± 3.21%) (Figure 3B and Additional file 3). Additional subsets of infected cells within the epithelium displayed positivity for CD163 (septum-E70: 8.33 ± 14.43%, septum-BEL18: 6.48 ± 5.78%, turbinate-E70: 12.54 ± 11.38%, turbinate-BEL18: 13.40 ± 12.99%), Sn (only identified in the septum-BEL18: 13.33 ± 23.09%), vimentin (septum-E70: 18.89 ± 20.09%, septum-BEL18: 22.22 ± 19.25%, turbinate-E70: 10.44 ± 11.17%, turbinate-BEL18: 30.42 ± 3.41%), and MHCII (only identified in the septum-E70: 4.76 ± 8.25%). Despite their presence, these subsets constituted a minor proportion of the total number of infected cells and exhibited a variability across individual animals (Figure 3B and Additional file 3). Infected cells within the epithelium expressing CD14 and vWF were absent.

Focusing on the lamina propria, a considerable proportion of the infected cells were found to be vimentin-positive (septum-E70: 93.50 ± 11.27%, septum-BEL18: 100%, turbinate-E70: 93.88 ± 10.60%, turbinate-BEL18: 90.00 ± 17.32%) (Figure 3B and Additional file 3). Except for the E70-inoculated nasal septum, a statistically larger proportion of infected cells in the lamina propria was found to be positive for vimentin, CD163, and vWF, compared to those in the epithelium and submucosa (Figure 3B and Additional file 3). Notably, ASFV^+^vWF^+^ cells were exclusively identified within the lamina propria (septum-E70: 37.05 ± 5.20%, septum-BEL18: 43.54 ± 4.00%, turbinate-E70: 28.34 ± 13.63%, turbinate-BEL18: 35.41 ± 6.96%) (Figure 3B and Additional file 3).

In the submucosa, a significant proportion of the infected cells were found to be positive for vimentin (septum-E70: 100%, septum-BEL18: 85.71 ± 14.29%, turbinate-E70: 94.87 ± 8.88%, turbinate-BEL18: 82.96 ± 5.13%) and CD163 (septum-E70: 77.78 ± 19.25%, septum-BEL18: 79.50 ± 5.56%, turbinate-E70: 75.40 ± 29.39%, turbinate-BEL18: 73.59 ± 23.10%) (Figure 3B and Additional file 3). This region in the turbinates primarily exhibited infected cells positive for CD14 (turbinate-E70: 96.30 ± 6.42% and turbinate-BEL18: 90.24 ± 9.17%), followed by Sn (turbinate-E70: 57.78 ± 36.72% and turbinate-BEL18: 31.19 ± 7.84%). The septum displayed a marked decrease in their prevalence (CD14: Septum-E70: 41.67 ± 52.045, septum-BEL18: 18.10 ± 31.34%; Sn: Septum-E70: 19.44 ± 17.35%, septum-BEL18: 28.14 ± 0.75%). Infected cells within the submucosa were not positive for MHCII, cytokeratin or vWF (Figure 3B and Additional file 3).

### ASFV genotype I strain E70 mainly targets the basolateral side, while genotype II strain BEL18 mainly targets apical surfaces of respiratory epithelial cells

Given our observation of ASFV primarily targeting cytokeratin-positive epithelial cells in the nasal epithelium, we further investigated the infection patterns of the two ASFV strains in porcine respiratory epithelial cells (PoRECs), employing both apical and basolateral side-inoculation strategies. To determine whether intercellular junction (ICJ) influences ASFV infection, cells were treated with 25 mM EGTA before inoculation at the apical surface.

**Figure 4:**
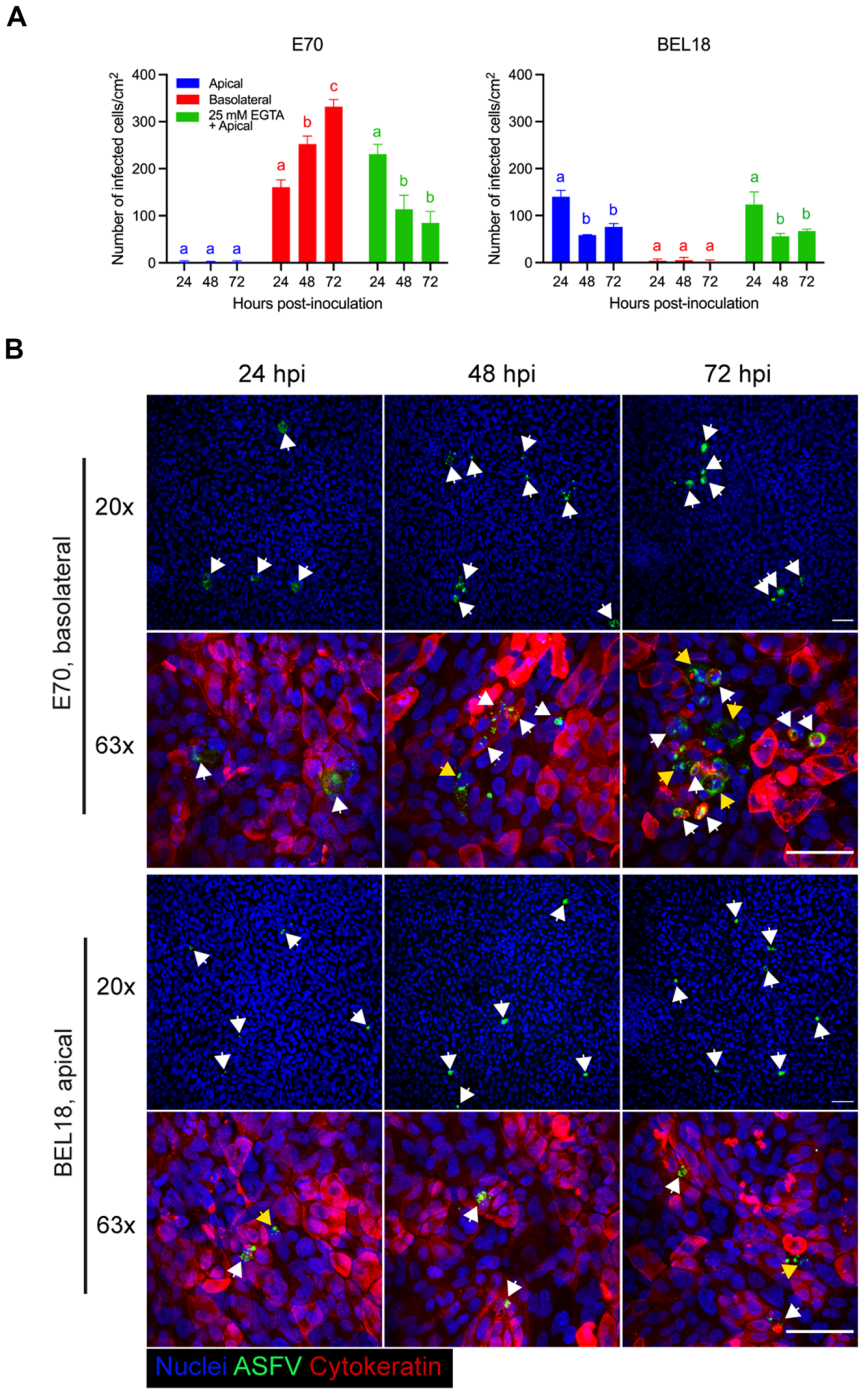
**Differential tropism of genotype I-E70 and genotype II-BEL strains in the respiratory epithelial cells.** **A** In vitro infection of E70 (left) and BEL18 (right) strains in respiratory epithelial cells. Cells were inoculated via apically, basolaterally, or apically after EGTA treatment. ASFV-infected cells were counted at 24, 48, and 72 hpi. Statistical significance was analyzed using two-way ANOVA followed by Tukey’s multiple comparison post hoc test. Different letters indicate significant differences (*p* < 0.05) between different time points. Data are presented as mean of three independent experiments ± SD. **B** Representative IF pictures of ASFV-infected PoRECs. Immunofluorescence staining was performed against ASFV p72 (green), cytokeratin (red), and nuclei (blue). The pictures show basolateral side infection of E70 (top) and apical side infection of BEL18 (bottom) in the respiratory epithelial cells for 72 h infection. Images were captured using either a 20× or 63× objective lens. Arrows indicate infected cells. In the images taken at 63× magnification, white arrows indicate cytokeratin^+^ infected cells, while yellow arrows point to cytokeratin^−^ infected cells. Scale bar: 50 μm.

Surprisingly, the two ASFV strains showed a different tropism of infection. For E70, the number of infected cells was significantly higher after inoculation at the basolateral surfaces (72 hpi: 331.48 ± 15.61 cells/cm^2^), than after apical inoculation (72 hpi: 2.22 ± 2.22 cells/cm^2^) (Figure 4A). The number of infected cells via basolateral inoculation significantly increased over incubation time (Figure 4A). Disruption of ICJ clearly influenced infection at 24 hpi (230.37 ± 21.27 cells/cm^2^). However, subsequent incubation resulted in a progressive decrease in infection (48 hpi: 113.70 ± 29.66 cells/cm^2^ and 72 hpi: 84.81 ± 24.48 cells/cm^2^) (Figure 4A). In contrast to E70, the number of BEL18-infected cells was significantly higher after inoculation at the apical surfaces (72 hpi: 76.30 ± 7.14 cells/cm^2^), compared to basolateral inoculation (1.85 ± 3.21 cells/cm^2^) (Figure 4A). BEL18 infection peaked at 24 hpi (140.00 ± 13.52 cells/cm^2^) and significantly decreased at 48 hpi (58.15 ± 1.28 cells/cm^2^) and slightly increased at 72 hpi (76.30 ± 7.14 cells/cm^2^, statistically not significant) (Figure 4A). Disruption of ICJ did not significantly increase the number of infected cells in apically inoculated PoRECs (Figure 4A).

IF staining of PoRECs revealed different features of infected cells by E70 and BEL18 strains. Following basolateral inoculation, E70-infected cells were found in close proximity of each other (plaque-wise appearance), whereas BEL18-infected cells, upon apical inoculation, appeared to be dispersed individually (Figure 4B). In the double IF staining against ASFV and the epithelial cell marker, cytokeratin, both ASFV^+^cytokeratin^+^ and ASFV^+^cytokeratin^−^ cells were identified (Figure 4B). Further characterization revealed that infected cells were found to be negative for both CD163 and vimentin (Additional file 4).

## Discussion

ASFV continues to pose a severe threat to global pig populations, causing substantial economic damage, disrupting food security, and giving issues with animal welfare. Among the several ASFV transmission routes, the oronasal route is a significant factor in understanding the viral spread, especially in animals with acute ASF [11]. The nasal mucosa, as an entry point for initial infection, as well as a reservoir of the virus, is a highly favorable pathway for transmission of several porcine respiratory viruses, such as swine influenza virus (SIV) and PRRSV [23, 24]. Experimental and field studies suggested the potential involvement of the upper respiratory system in the transmission of ASFV [11, 25, 26], however, ASFV infection in the nasal mucosa was not studied yet. In this study, we employed a well-established porcine nasal explant model [19], designed to mimic the highly multifaceted respiratory environment. We isolated two types of nasal tissue: the nasal septum and the nasal turbinates. These tissues were subsequently cultivated at the air–liquid interface and inoculated with genotype I strain E70 and genotype II strain BEL18.

We first analyzed the viability of nasal mucosa explants during in vitro cultivation. Our findings demonstrated that cells in the epithelium (EP) and the lamina propria (LP) maintained high levels of viability after 72 h of incubation. We observed an increase in the number of apoptotic cells within the submucosa (SM). However, these cells were mainly restricted at the edge of the tissue, suggesting the potential damage during the cutting of the explant. Grossly, the nasal explants appeared to be viable without substantial morphological change.

Our results, obtained via immunofluorescence staining for the major ASFV capsid protein p72 and monocyte/macrophage marker CD163, uncovered key aspects of ASFV infection within the nasal explant. Intriguingly, infections by both genotypes (genotype I strain E70 and genotype II strain BEL18) were discerned within the nasal septum and turbinates. Over the incubation period after inoculation, we noted a rise in the total number of infected cells in both nasal tissues for both strains. Although the difference between E70 and BEL18 strains was not significant, we observed a higher number of infected cells in the turbinate explant compared to the septum explant. We additionally measured the thickness of the septum and turbinate explants, and surprisingly, the septum was significantly thicker than the turbinate, which appeared to be due to the thicker submucosal layer (Additional file 5). Despite this, our results showed that more infected cells were found in the turbinates, suggesting that the turbinate tissue exhibits a better susceptibility to ASFV. When comparing the two ASF genotypes, it became clear that, in the epithelial cell layer, two times more cells were infected with genotype II compared to genotype I.

Our comprehensive investigation into the distribution of ASFV infection across different areas in the nasal mucosa revealed insightful results. Analysis of the infected cells in three distinct regions (epithelial cell layer, lamina propria, and submucosa), revealed that the lamina propria is a preferential site for ASFV replication, irrespective of the viral strain. The infected cells were found in clusters, predominantly in the EP and LP implying a spread in a cell-associated way. Although it is generally accepted that the main target cells of ASFV are monocytes and macrophages [27], a significant number of CD163-negative infected cells were found across the different regions of the nasal mucosa. Further phenotypic characterization of these infected cells revealed that different types of cells in the nasal mucosa are susceptible to ASFV. A significant fraction of virus-infected cells within the epithelial cell layer was found to be cytokeratin-positive epithelial cells. In the LP, we found that many infected cells were positive for vimentin, CD163, MHCII, and vWF, indicating that the major target cells in this tissue area are macrophage-like and endothelial cells. In the submucosa, vimentin and CD163-positive infected cells were found, pointing in the direction of macrophages as targets. The prevalence of CD14 and Sn positive cells in the population of infected cells varied depending on the specific nasal region, virus strain, and individual animals. ASFV primarily targets macrophages and monocytes. The scavenger receptor, CD163 is expressed in cells of the monocytic lineage and its role as an ASFV receptor has been proposed [28]. However, recent findings suggest that it may not be an essential factor, given that CD163 knockout pigs can still be infected by the virus [29]. In addition, many studies demonstrated that ASFV can also infect other cell types such as dendritic cells, hepatocytes, renal tubular epithelial cells, endothelial cells, and some established cell lines with less efficiency [30–35]. Our finding of infected cells with various phenotypes in the nasal mucosa is in agreement with these findings, highlighting the versatile cellular tropism of ASFV. While our study highlighted the wide cell tropism of ASFV, it remains essential for future studies to determine if these cell types can produce infectious virus.

Building further upon our findings, which revealed a significant proportion of cytokeratin-positive infected cells in the epithelium, we expanded our investigation by inoculating two ASFV strains in the respiratory epithelial cells isolated from the nasal tissue (PoRECs). Intercellular junctions (ICJ) play a pivotal role in maintaining the integrity and polarity of the ciliated pseudostratified columnar epithelium by providing a distinct barrier between the apical and basolateral domains of the cell. Certain viruses, such as respiratory syncytial virus, hepatitis A virus, and simian virus 40, display a preference for infecting via the apical surface of the cells [36–38], while other viruses, such as adenoviruses, demonstrate a tropism for the basolateral side of the cells [39]. Our findings suggest a genotypic variation in the mechanism of ASFV infection, especially in their preference for apical or basolateral surfaces of PoRECs. ASFV genotype I, E70, was found to primarily target the basolateral surface of the epithelial cells. Interestingly, the disruption of ICJ with EGTA influenced the infection rates at the early stages (24 hpi), however, subsequent incubation resulted in a progressive decrease in infection. The difference between these two situations (basolateral versus EGTA/apical inoculation), may be related to the repair of the ICJ upon culturing the cells. In contrast, the genotype II, BEL18 infection, showed a preference for the apical surface. The number of infected cells peaked at 24 h after apical inoculation but decreased in further incubation period. Moreover, disruption of ICJ did not significantly change the infection rate, demonstrating that the apical infection mechanism of the BEL18 strain is independent of the ICJ integrity. This observation aligns with our explant results, showing that twice as many cells were infected by the BEL18 compared to the E70 in the epithelial cell layer. The preferential targeting of the apical surface by genotype II could potentially explain its ability to infect more cells rapidly. A recent study on equine herpes virus 1 demonstrated that restriction of infection via apical inoculation was overcome by disruption of ICJ with EGTA treatment, subsequently leading to virus infection at the basolateral cell surfaces [40]. This aligns with our findings in E70 infection that this strain favors infection via the basolateral surfaces, which was facilitated after EGTA treatment, while there was no effect on BEL18 strain, which prefers infection via the apical surface of the cells. Taking all these results together, one may conclude that the E70 is likely less efficient in infecting epithelial cells in healthy pigs. It is likely that replication may only be facilitated when the epithelial cell layer is compromised, potentially due to exposure to toxins and proteases from other pathogens, or even the virus itself [41–44]. Despite the genotype II BEL18 strain’s tendency for apical-side infection providing a higher initial transmissibility than genotype I, the infection rate reduction 48 hpi indicates a potential limitation to the replication of genotype II within the epithelial cells. The mechanism behind possible antiviral responses in the nasal mucosa should be investigated in the future study.

We also identified cytokeratin-negative infected cells in our PoRECs. Additional triple IF staining against ASFV, vimentin, and CD163 revealed that infected cells are neither mesenchymal/fibroblast cells nor monocytes/macrophages. This suggests that these cytokeratin-negative infected cells are probably non-differentiated epithelial cells. In the future study, an approach with various epithelial cell markers is necessary to better characterize these cells. Based on these findings, one may conclude that genotype I and genotype II use different receptors to enter the nasal mucosa. Future work will focus on the identification of these receptors.

In summary, the present study is the first that investigated ASFV infection in the nasal mucosa. The findings from our ex vivo model have revealed that ASFV demonstrates a broad cell tropism in the nasal mucosa. Furthermore, our in vitro model using primary respiratory epithelial cells highlights that epithelial cells are susceptible to ASFV and that the two genotypes use different surfaces for infection: genotype I basolaterally and genotype II apically. While our study provides valuable insights into the molecular and cellular aspects of ASFV infection within the nasal mucosa, they have inherent limitations as they cannot fully replicate the complex environment within a living organism. Therefore, future in vivo studies will be crucial for gaining a more comprehensive understanding of ASFV pathogenesis in the nasal mucosa at early timepoints in infection. The knowledge gained from our current research will significantly facilitates the interpretation of subsequent in vivo results. Our study shed new light on the pathogenesis of ASFV in the respiratory system and emphasizes the possible role of the nasal route for ASFV transmission.

### Supplementary Information

**Additional file 1. Reactivity of selected cell markers in the lung and nasal tissue sections.** Immunofluorescence staining for the selected cell markers was performed on the lung and nasal tissue sections. Reactivity signal is presented in plus and minus symbols. Abbreviations—Mo: monocyte, Mf: macrophage, Mc: myeloid cell, APC: antigen presenting cell, Ep: epithelial cell, MSc: mesenchymal cell, Fi: fibroblast, En: endothelial cell.

**Additional file 2. Immunofluorescence staining of the lung tissue section.** All the cell markers were positive in the lung tissue except vWF. Cell markers (red), and nuclei (blue). Scale bar: 50 μm.

**Additional file 3. Percentage of cell marker positive cells within ASFV-infected cells.** Quantification and calculation were made in three regions in the nasal septum (A) and turbinate (B) explants: epithelium (EP), lamina propria (LP), and submucosa (SM). Septum-E70: E70 inoculated septum explant, septum-BEL18: BEL18 inoculated septum explant, turbinate-E70: E70 inoculated turbinate explant, turbinate-BEL18: BEL18 inoculated turbinate explant. Values are presented as mean value of three animals ± SD.

**Additional file 4. Characterization of ASFV infected PoRECs.** Triple immunofluorescence staining for ASFV p72 (green), vimentin (red), and CD163 (teal) was performed on respiratory epithelial cells after inoculation with E70 and BEL18 strains. Cell nuclei were presented in blue. Scale bar: 50 μm.

**Additional file 5. Measurement of the nasal explant thickness.** (A) Areas measured in the nasal septum and turbinate explants. (B) The width and thickness of the nasal explants were measured. The total thickness of the septum tissue was significantly higher than that of the turbinate tissue. (C) The thickness of different areas in the septum and turbinate explants was measured. The submucosa was significantly thicker in the septum compared to that of the turbinates. Statistical analysis for (B) and (C) was performed using two-way ANOVA followed by Šidák’s multiple comparison test (****p < 0.0001).

## Data Availability

The data that support the findings of this study are available on request from the authors, under the reasonable request.
