# Supplementary material for: Differential infection behavior of African swine fever virus (ASFV) genotype I and II in the upper respiratory tract
Source: Vet Res. 2023 Dec 15;54:121. doi: 10.1186/s13567-023-01249-8 (PMC10725007; doi:10.1186/s13567-023-01249-8)
Supplement: Supplementary file 1 — Additional file 1. Reactivity of selected cell markers in the lung and nasal tissue sections. Immunofluorescence staining for the selected cell markers was performed on the lung and nasal tissue sections. Reactivity signal is presented in plus and minus symbols. Abbreviations—Mo: monocyte, Mf: macrophage, Mc: myeloid cell, APC: antigen presenting cell, Ep: epithelial cell, MSc: mesenchymal cell, Fi: fibroblast, En: endothelial cell. [file 13567_2023_1249_MOESM1_ESM.docx]

|  | | Reactivity | |
| --- | --- | --- | --- |
| Markers | Cell types | Lung | Nasal |
| CD163 | Mo, Mf | ++++ | ++ |
| CD14 | Mo, Mf | + | + |
| SWC3 | Mc | +++ | - |
| Sn | Mf | +++++ | + |
| CD1c | APC | + | - |
| Cytokeratin | Ep | +++++ | +++++ |
| Vimentin | MSc, Fi | +++++ | +++++ |
| MHCII | APC | +++ | ++ |
| vWF | En | - | +++++ |
