# Supplementary figures and images for: Differential infection behavior of African swine fever virus (ASFV) genotype I and II in the upper respiratory tract
Source: Vet Res. 2023 Dec 15;54:121. doi: 10.1186/s13567-023-01249-8 (PMC10725007; doi:10.1186/s13567-023-01249-8)

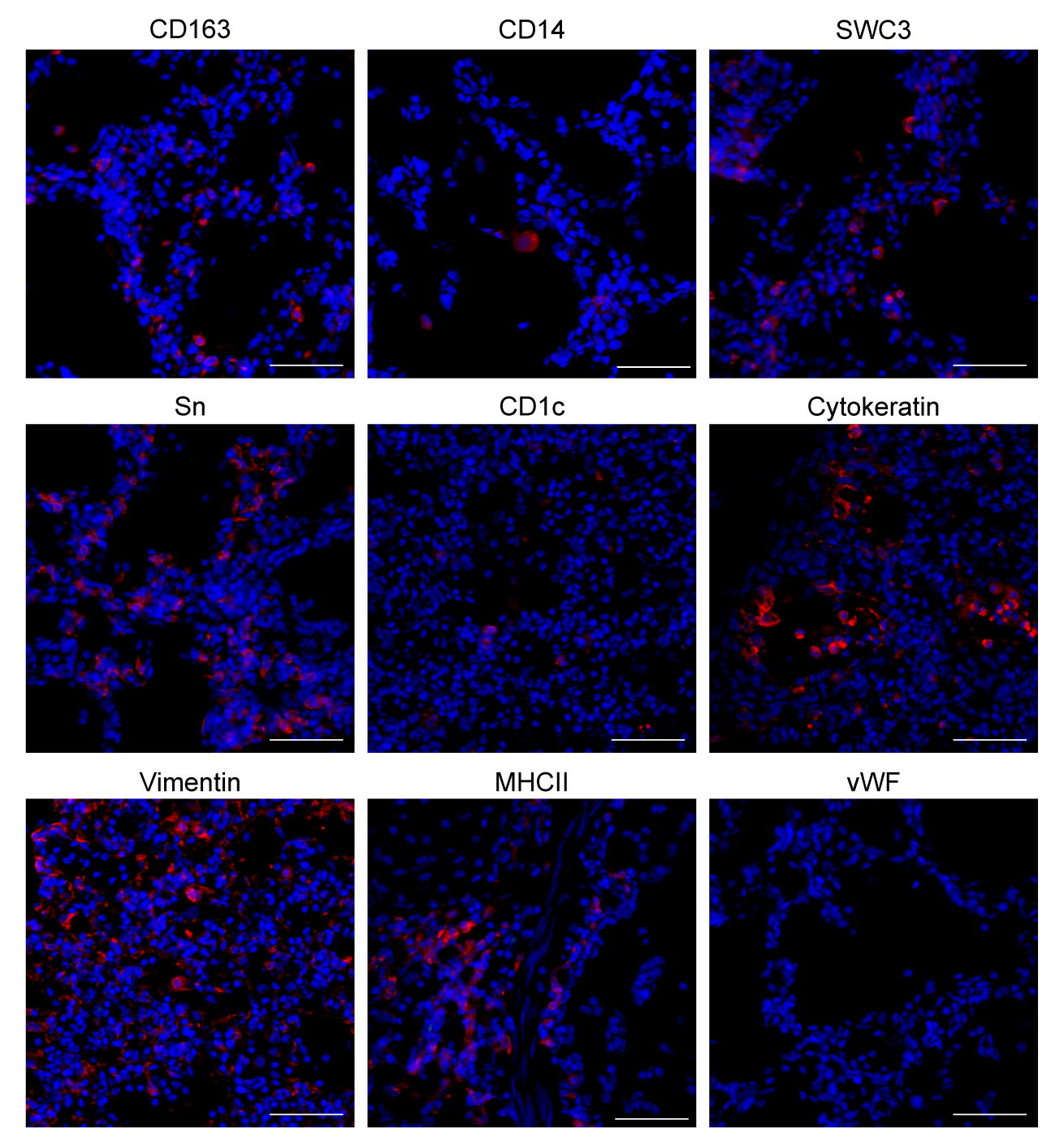

Supplement: Supplementary file 2 — Additional file 2. Immunofluorescence staining of the lung tissue section. All the cell markers were positive in the lung tissue except vWF. Cell markers (red), and nuclei (blue). Scale bar: 50 μm. [file 13567_2023_1249_MOESM2_ESM.tif]

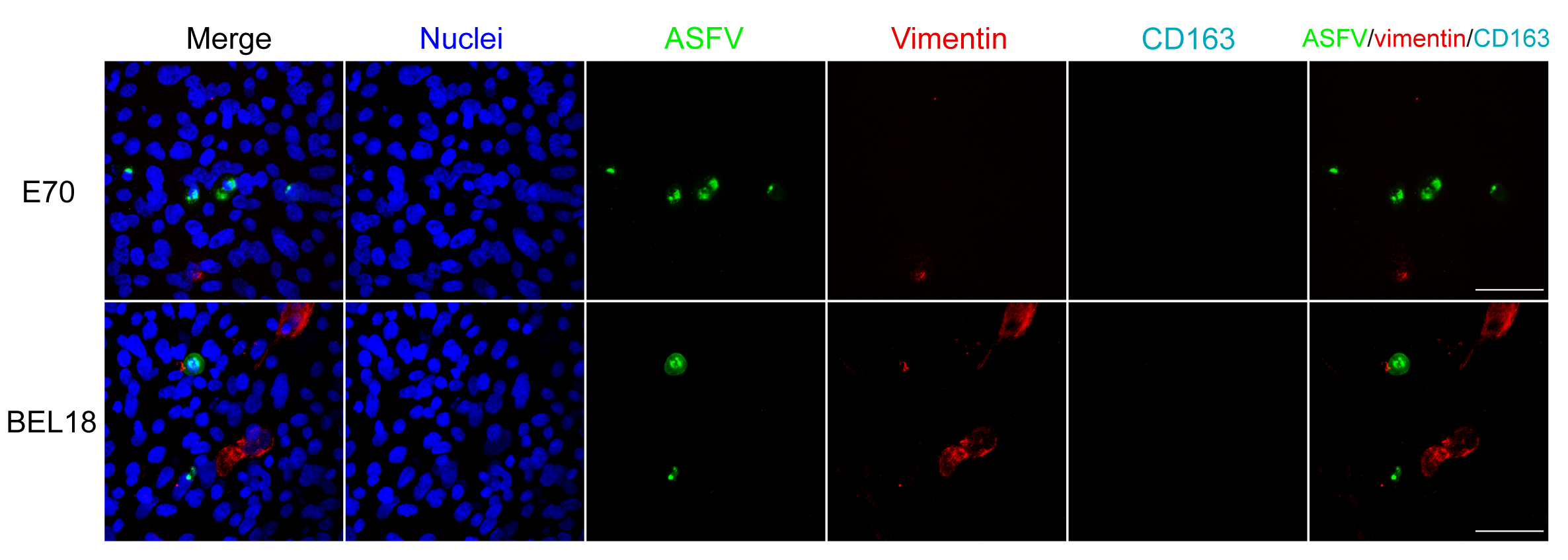

Supplement: Supplementary file 4 — Additional file 4. Characterization of ASFV infected PoRECs. Triple immunofluorescence staining for ASFV p72 (green), vimentin (red), and CD163 (teal) was performed on respiratory epithelial cells after inoculation with E70 and BEL18 strains. Cell nuclei were presented in blue. Scale bar: 50 μm. [file 13567_2023_1249_MOESM4_ESM.tif]

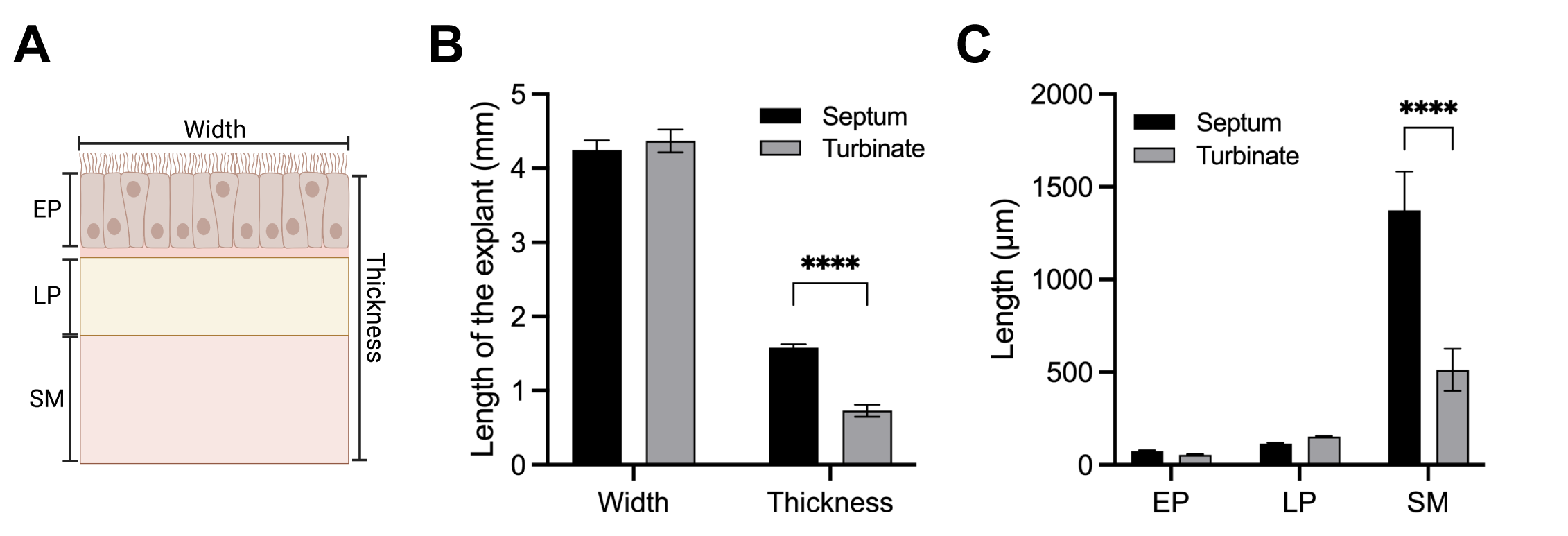

Supplement: Supplementary file 5 — Additional file 5. Measurement of the nasal explant thickness. (A) Areas measured in the nasal septum and turbinate explants. (B) The width and thickness of the nasal explants were measured. The total thickness of the septum tissue was significantly higher than that of the turbinate tissue. (C) The thickness of different areas in the septum and turbinate explants was measured. The submucosa was significantly thicker in the septum compared to that of the turbinates. Statistical analysis for (B) and (C) was performed using two-way ANOVA followed by Šidák’s multiple comparison test (****p < 0.0001). [file 13567_2023_1249_MOESM5_ESM.tif]
