# Supplementary material for: Differential infection behavior of African swine fever virus (ASFV) genotype I and II in the upper respiratory tract
Source: Vet Res. 2023 Dec 15;54:121. doi: 10.1186/s13567-023-01249-8 (PMC10725007; doi:10.1186/s13567-023-01249-8)
Supplement: Supplementary file 3 — Additional file 3. Percentage of cell marker positive cells within ASFV-infected cells. Quantification and calculation were made in three regions in the nasal septum (A) and turbinate (B) explants: epithelium (EP), lamina propria (LP), and submucosa (SM). Septum-E70: E70 inoculated septum explant, septum-BEL18: BEL18 inoculated septum explant, turbinate-E70: E70 inoculated turbinate explant, turbinate-BEL18: BEL18 inoculated turbinate explant. Values are presented as mean value of three animals ± SD. [file 13567_2023_1249_MOESM3_ESM.docx]

(A)

| Cell markers | Septum-E70 | | | | Septum-BEL18 | | | |
| --- | --- | --- | --- | --- | --- | --- | --- | --- |
|  | EP | LP | SM | EP | | LP | SM |  |
| CD163 | 8.33 ± 14.43 | 38.77 ± 23.42 | 77.78 ± 19.25 | 6.48 ± 5.78 | | 46.02 ± 22.34 | 79.50 ± 5.56 |  |
| CD14 | 0 | 29.33 ± 13.60 | 41.67 ± 52.04 | 0 | | 12.68 ± 9.54 | 18.10 ± 31.34 |  |
| Sn | 0 | 23.74 ± 2.19 | 19.44 ± 17.35 | 13.33 ± 23.09 | | 19.61 ± 8.01 | 28.14 ± 0.75 |  |
| MHCII | 4.76 ± 8.25 | 40.22 ± 33.21 | 52.78 ± 41.11 | 0 | | 49.47 ± 5.10 | 0 |  |
| Cytokeratin | 93.33 ± 11.55 | 2.08 ± 3.61 | 0 | 85.56 ± 17.11 | | 0.93 ± 1.60 | 0 |  |
| Vimentin | 18.89 ± 20.09 | 93.50 ± 11.27 | 100.00 | 22.22 ± 19.25 | | 100 | 85.71 ± 14.29 |  |
| vWF | 0 | 37.05 ± 5.20 | 0 | 0 | | 43.54 ± 4.00 | 0 |  |

(B)

| Cell markers | Turbinate-E70 | | | Turbinate-BEL18 | | |
| --- | --- | --- | --- | --- | --- | --- |
|  | EP | LP | SM | EP | LP | SM |
| CD163 | 12.54 ± 11.38 | 71.04 ± 14.41 | 75.40 ± 29.39 | 13.40 ± 12.99 | 56.21 ± 28.50 | 73.59 ± 23.10 |
| CD14 | 0 | 33.96 ± 3.55 | 96.30 ± 6.42 | 0 | 35.74 ± 4.46 | 90.24 ± 9.17 |
| Sn | 0 | 27.23 ± 7.47 | 57.78 ± 36.72 | 0 | 23.45 ± 1.35 | 31.19 ± 7.84 |
| MHCII | 0 | 29.57 ± 24.60 | 0 | 0 | 49.19 ± 15.88 | 0 |
| Cytokeratin | 96.30 ± 6.42 | 2.25 ± 2.39 | 0 | 98.15 ± 3.21 | 0.71 ± 1.23 | 0 |
| Vimentin | 10.44 ± 11.17 | 93.88 ± 10.60 | 94.87 ± 8.88 | 30.42 ± 3.41 | 90.00 ± 17.32 | 82.96 ± 5.13 |
| vWF | 0 | 28.34 ± 13.63 | 0 | 0 | 35.41 ± 6.96 | 0 |
